# Assessing the Martini Force Field for Modeling Polyolefin Nanoplastics near Lipid Membranes

**DOI:** 10.1021/acs.jpcb.6c02507

**Published:** 2026-06-15

**Authors:** Anderson D. S. Duraes, Caleb Liu, Wenlin Zhang

**Affiliations:** Department of Chemistry, 3728 Dartmouth College, 41 College Street, Hanover, New Hampshire 03755, United States

## Abstract

We present an improved Martini-type coarse-grained (CG) model for polyethylene (PE) nanoplastics and benchmark its performance against three existing Martini PE models from the Martini 2 and Martini 3 force fields. While current Martini models reproduce conformational statistics for molten PE, the PE chains do not crystallize below experimental melting temperatures. With improved bonded interactions, our CG PE chains exhibit melt properties and semicrystalline morphologies consistent with all-atom (AA) simulations and experimental data. Using our improved model, we generate semicrystalline PE nanoplastics (NPLs) at body temperature (310 K) in agreement with the AA reference, whereas NPLs from current Martini PE models remain amorphous. We further investigate the interaction of semicrystalline PE NPLs with a POPC lipid membrane in the Martini framework. The membrane exhibits unphysical behavior similar to that observed for amorphous NPLs, bending toward and mixing with the nanoplastic. When embedded within the membrane core, the semicrystalline Martini-type NPL spreads laterally and dissolves, losing its crystalline domains, whereas the all-atom NPL remains intact, with alkane–membrane interactions promoting further crystallization. These results indicate that improving the PE model alone is insufficient and that refining the membrane model is also required to accurately describe polyolefin nanoplastic–membrane interactions in the Martini force field.

## Graphical abstract

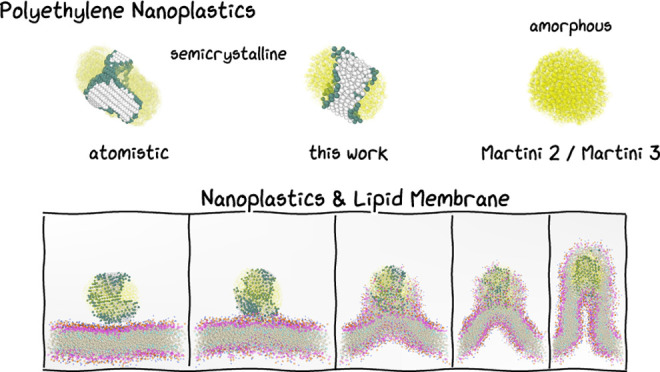

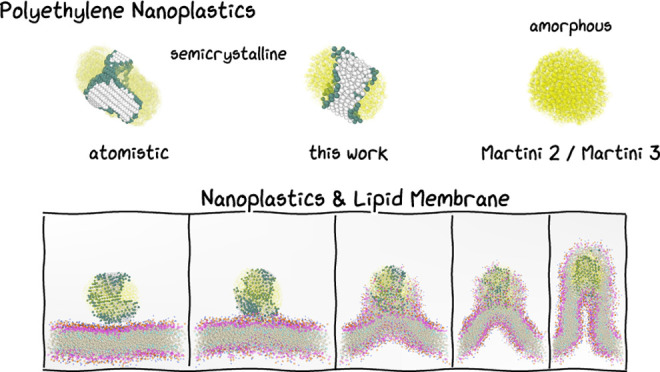

## 1 Introduction

Micro- and nanoplastics have emerged as pervasive environmental contaminants, raising growing concern about their potential impacts on human health.^1–5^ Among them, polyethylene (PE) is one of the most abundant polymer types, owing to its water insolubility and high resistance to degradation, which underpin its widespread use in packaging and consumer products.^6–10^ A recent atomistic molecular dynamics (MD) study^11^ indicates that bare PE nanoplastics are unlikely to translocate through a cell membrane on physiologically relevant time scales and complements experimental observations showing that PE microplastics do not penetrate the hydrophobic core of a model human plasma cell membrane.^12^ However, interactions between bare nanoplastics and surrounding biomolecules can lead to the formation of biocoronae, which may facilitate cellular uptake when the chemical attraction between a biocorona and the membrane is sufficiently strong.^13–15^

Despite extensive experimental work investigating the effects of micro- and nanoplastics on living systems, the molecular mechanisms governing their interactions with cell membranes are not yet fully understood.^16–21^ Atomistic MD simulations provide a powerful complementary approach for elucidating these mechanisms, but they are inherently limited to relatively small system sizes and short time scales. To overcome these limitations, coarse-grained models, such as the Martini force field, are commonly employed to access larger spatial and temporal scales. The Martini force field was specifically developed for biomolecular simulations and is widely used to reduce computational cost while retaining essential molecular features.^22,23^ Nevertheless, this computational efficiency does not always translate into an accurate description of biomolecular systems. In particular, although the Martini force field continues to evolve, it has been shown to produce brittle lipid membranes, nonphysical polymer–membrane interactions, and polymer morphologies in which PE crystallization is not observed.^11,24–26^

In this work, we propose a simple Martini-type PE model to better reproduce experimental morphologies. We use the all-atom CHARMM36 force field as a reference,^27–29^ as it is widely used in biomolecular simulations, reproduces experimental PE data across different degrees of polymerization and temperatures, and is capable of generating PE crystals within MD time scales.^30,31^ In addition, we benchmark our PE model against three other PE models available in Martini. These other models are designed to reproduce polymer melt statistics, such as end-to-end distances, radius of gyration, and density, but do not account for nucleation or crystallization of PE chains. As reported in ref 11, this results in PE nanoplastics with nonphysical morphologies, forming liquid droplets rather than the semicrystalline structures observed in experiments and all-atom (AA) simulations.^31,32^ Our proposed Martini-type model reproduces both PE melt statistics and crystallization, which occurs via homogeneous nucleation, in good agreement with the AA reference. However, simulating this new PE model with a POPC membrane still leads to nonphysical attraction between the nanoplastic and the membrane. Moreover, simulations of semicrystalline PE nanoplastics within the membrane hydrophobic core reveal additional mismatches: while membranes induce further crystallization of PE in AA simulations, they dissolve the PE nanoplastic in Martini. We conclude that the Martini membrane parameters require further refinement to match AA simulations and to predict more realistic polymer–lipid interactions.

## 2 Methods

In this section, we describe the simulation parameters and details of the PE systems. We also present our Martini-type PE model parameters and details of polymer chain statistics calculations. The procedure for forming semicrystalline PE nanoplastics and the calculation of the potential of mean force between our PE model and a POPC membrane immersed in water are described in Results and Discussion (Sec 3.3). All simulations are carried out using GROMACS 2023.3 ^33^ with periodic boundary conditions applied in all directions.

### 2.1 AA Model

We construct ten polyethylene oligomer samples with chain lengths ranging from 10 to 100 monomers, in increments of 10 monomers, using the *Polymer Builder* module in CHARMM-GUI^30,34^ and the all-atom CHARMM Generalized Force Field (CGenFF).^29^ Each sample consists of 200 chains in a cubic simulation box. The systems are equilibrated under NPT conditions for 10^8^ steps using the stochastic velocity rescaling thermostat^35^ with a time coupling constant of 1 ps at 450 K, together with the Berendsen barostat^36^ with a time coupling constant of 2 ps at 1 bar. Postequilibration NPT simulations replace the barostat with the Parrinello–Rahman barostat,^37^ applied every 20 ps, and are run for 1.5 × 10^8^ steps unless stated otherwise. All atomistic simulations are performed with a time step of 0.002 ps, and data are collected during postequilibration runs.

Additionally, samples with 50 (PE50) and 100 monomers (PE100) are simulated at fixed temperatures ranging from 410 to 500 K, in increments of 5 K, to investigate PE conformations as a function of temperature. For each temperature, we apply the same equilibration and postequilibration protocols as used at 450 K.

### 2.2 Existing Martini PE Models

We construct the same PE oligomers as in the AA model for three Martini force-field variants using *Polyply*: Martini 2, Martini 3­(v1), and Martini 3­(v2).^38^ The corresponding force-field parameters are reported in ref  ^39^. The main differences among these models lie in the functional forms used to represent bonded interactions, specifically angles and dihedrals, and in their associated parameters. The Martini 2 model^40^ employs a cosine-based angle potential (G96 angle),^41,42^

1 $$V_{a,G96}\left(\theta_{ijk}\right)=\frac{1}{2}\;\kappa_{\theta ,G96}\;{[\cos\left(\theta_{ijk}\right)-\cos\left(\theta_{ijk,0}\right)]}^{2}$$

where κ_θ,G96_ is the angle force constant, θ_*ijk*_ is the angle formed by three consecutive coarse-grained (CG) beads *i*, *j*, and *k* with vertex at *j*, and θ_*ijk*,0_ is the corresponding reference angle. This angle potential is used together with a combined bending-torsion (CBT) potential for dihedral interactions, as proposed for polymer melts by Bulacu and van der Giessen.^43^ The Martini 3­(v1) model^38^ also uses the CBT potential but employs a harmonic angle potential for bending vibrations,

2 $$V_{a}\left(\theta_{ijk}\right)=\frac{1}{2}\;\kappa_{\theta }\;{\left(\theta_{ijk}-\theta_{ijk,0}\right)}^{2}$$

where the terms are defined analogously to those in eq 1. The force constant in the G96 angle potential is a temperature-dependent function of κ_θ_ (see Sec. III of the Supporting Information). The Martini 3­(v2) model,^38^ in turn, adopts the G96 angle term (eq 1) without an explicit dihedral potential. All these models utilize the apolar C1 Martini bead type to represent two monomers of the AA model (Figure 1). These C1 beads are uncharged, and their nonbonded interactions consist solely of the 12–6 Lennard-Jones (LJ) potential,

3 $$V_{LJ}\;\left(r_{ij}\right)=4\varepsilon \;\left[{\left(\frac{\sigma }{r_{ij}}\right)}^{12}-{\left(\frac{\sigma }{r_{ij}}\right)}^{6}\right]$$

where *r*_*ij*_ is the distance between nonbonded beads *i* and *j*. The LJ parameters are a common σ = 0.47 nm and ε = 3.5 kJ/mol for Martini 2,^22^ and 3.39 kJ/mol for the two Martini 3 versions.^23^ Additionally, the C1 bead has a mass of 54 u (unified atomic mass unit) in Martini 2 to reproduce PE melt densities from the AA model, whereas the Martini 3 models assign a mass of 72 u.

**Figure 1:**
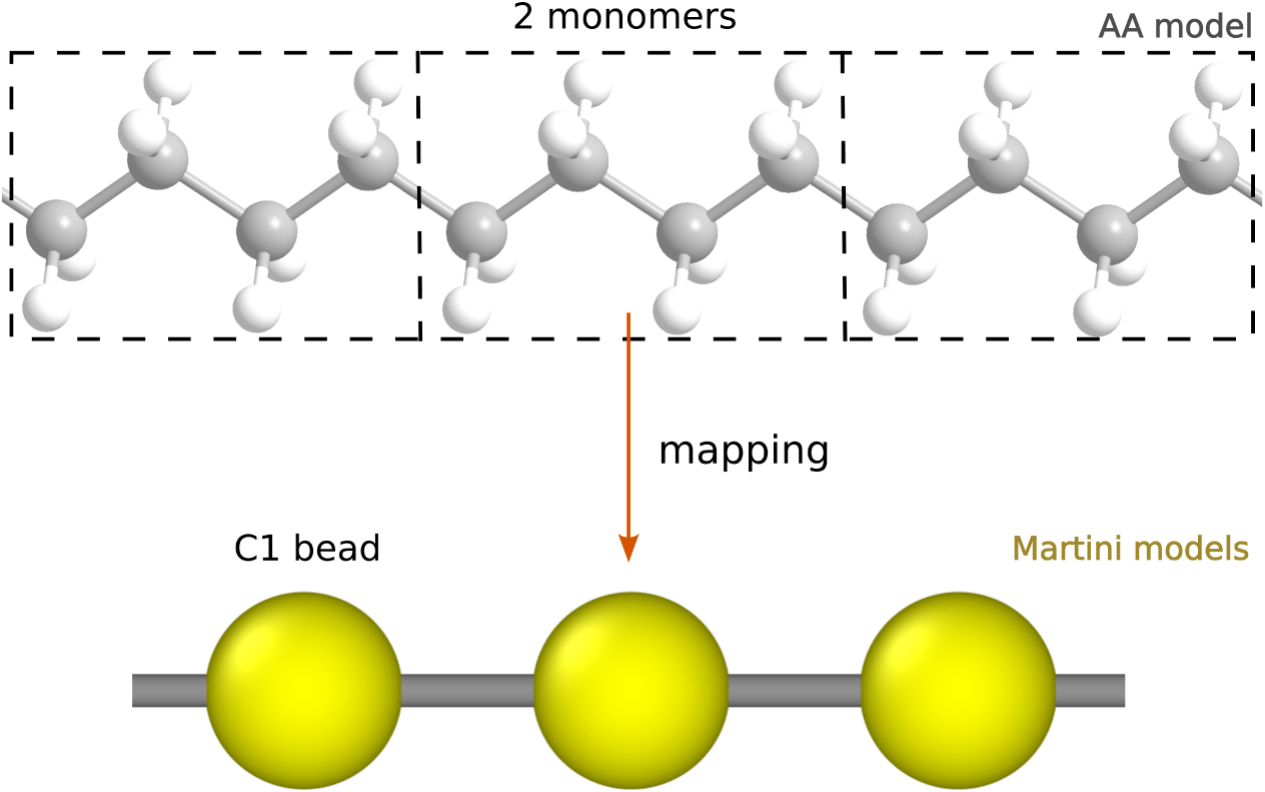
Two polyethylene (PE) monomers are mapped to a single apolar C1 bead (yellow) from the Martini force field. Carbon atoms shown as gray spheres, hydrogen atoms as white spheres, and bonds as gray rods.

We apply the same equilibration and postequilibration protocols as in the AA model but with a time step of 0.02 ps. In addition, we perform simulations at temperatures ranging from 410 to 500 K for coarse-grained polymers with 25 (PE25 (CG)) and 50 (PE50 (CG)) beads, corresponding to the same polymers in the AA model. The label (CG) is used to differentiate the coarse-grained models from their AA counterparts.

### 2.3 Polymer Chain Statistics

#### 2.3.1 Root-Mean-Square End-to-End Distance

The root-mean-square (RMS) end-to-end distance of a polymer quantifies the average separation between its two ends and is computed as^44,45^

$$\langle R^{2}\rangle^{1/2}=\sqrt{\langle R_{s}\cdot R_{s}\rangle_{s,t}}$$ 4

where **R**_*s*_ is the vector connecting the first unit to the last unit of the backbone of polymer *s*, averaged over polymer chains within each time frame and over time frames *t*.

#### 2.3.2 Root-Mean-Square Radius of Gyration

Root-mean-square (RMS) radius of gyration measures the spatial extent of a polymer chain around its center of mass *M* and is calculated as^31^

5 $$\langle R_{g}^{2}\rangle^{1/2}=\sqrt{\langle \mathrm{Tr}\left(S_{g}\right)\rangle_{s,t}}$$

where Tr denotes the trace of the mass gyration tensor for a polymer *s*

6 $$S_{g}=\frac{1}{\underset{i}{\sum }m_{i}}\underset{i}{\sum }m_{i}\;\left(r_{Mi}⊗r_{Mi}\right)$$

with *m*_*i*_ the mass of element *i* of the polymer *s*, **r** *_Mi_* the vector connecting *M* to *i* and the symbol ⊗ denoting outer product. The trace of **S**_*g*_ in eq 5 is averaged over polymer chains within each time frame and over time frames *t*.

#### 2.3.3 Persistence Length

The persistence length *l*_*p*_ characterizes the intrinsic stiffness of a polymer and is defined from the decay of orientational correlations along backbone bonds separated by *n*,^44,45^

7 $$\langle {\mathrm{b̂}}_{s}\left(0\right)\cdot {\mathrm{b̂}}_{s}\left(n\right)\rangle_{s}=\langle \cos\left[\theta_{s}\left(n\right)\right]\rangle_{s}\approx \exp\left(\frac{-nl_{b}}{l_{p}}\right)=\exp\left(\frac{-n}{N_{p}}\right)$$

where **b̂** *_s_* is the unit bond vector connecting consecutive backbone elements of a polymer *s*, oriented from the first to the last element along the backbone, θ_*s*_(n) is the angle between bond vectors separated by *n*, *l*_*b*_ is the average backbone bond length, and *N*_*p*_ = *l*_*p*_/*l*_*b*_ is the persistence length expressed in units of bonds. For each time frame, the orientational correlation function is computed over all possible bond pairs with separation *n*, averaged over polymer chains, and fitted to extract *l*_*p*_; the reported persistence length is then obtained by averaging over time frames.

#### 2.3.4 Order Parameter

To identify crystalline atoms *c*, we adopt the same criterion as in refs 11, 31 . For each atom *c*, we consider unit tangent vectors **t̂**_*s*_ connecting consecutive monomers along the backbone of each polymer *s*, whose coordinates lie within a radial distance of 1.08 nm from atom *c*. Using these vectors, we compute the local nematic order tensor^11,31,46^

$$\left\langle Q_{c}\right\rangle =\frac{3}{2}\langle {\mathrm{t̂}}_{s}⊗{\mathrm{t̂}}_{s}\rangle_{\mathrm{t̂}}-\frac{1}{2}I$$ 8

where ⟨⟩_**t̂**_ denotes an ensemble average over all such tangent vectors, and **I** is the 3 × 3 identity matrix. The largest eigenvalue *q*_*c*_ of **Q**_*c*_ (eq 8) ranges from 0 (isotropic) to 1 (perfect uniaxial order).^31,46^ Atoms with *q_c_* ≥ 0.8 are classified as crystalline.^11,31^ Unless otherwise stated, atoms with *q_c_* ≥ 0.9 are shown in white and those with 0.8 ≤ *q_c_* < 0.9 in dark green. The fraction of crystalline atoms relative to the total number of atoms defines the crystallinity χ.

#### 2.3.5 Interchain Radial Distribution Function

To compare the structure of PE polymer melts and crystals independently of the polymer mapping, we compute the interchain radial distribution function of backbone atoms,^46^

9 $$g\left(r\right)=\frac{N_{shell}\left(r\right)}{\rho V_{shell}\left(r\right)}$$

where *N_shell_* (*r*) is the number of interchain backbone atom pairs with separation *r* counted within a spherical shell, ρ is the number density of interchain backbone atom pairs in the system, and *V_shell_* (*r*) is the shell volume.

### 2.4 Our Martini-Type PE Model

We map two consecutive PE monomers to a single C1 bead (see Figure 1), the same mapping used in existing PE models (Sec 2.2). The C1 nonbonded interactions are taken from the Martini 2 force field. To extract the bonded force-field potentials and parameters, we apply the same mapping to the AA model and analyze the resulting bond stretching, bond angle, and dihedral angle distributions in comparison with the available PE models. These distributions are provided in Sec. I of the Supporting Information. We adopt the rotational isomeric states (RIS) notation and angle ranges reported in Table 2 of ref 47: *trans* (*T*), *gauche* ^–^ (*g*) and *gauche* ^+^ (*G*). The state *X* denotes dihedral angles that do not fall within the *T*, *G*, or *g* states. For the AA-to-CG mapping, the C1 bead position is taken as the first carbon atom of each two-monomer unit. Choosing a different carbon atom within the two-monomer unit does not change the statistical properties of the mapped AA model. A comparison of different AA-to-CG mappings is available in Sec. I of the SI.

Current PE models similarly smear out the conformational states observed in the bond stretching distribution of the AA model by using a harmonic potential,

10 $$V_{b}\left(b_{ij}\right)=\frac{1}{2}\;\kappa_{b}\;{\left(b_{ij}-b_{ij,0}\right)}^{2}$$

where κ_*b*_ is the bond force constant, *b*_*ij*_ is the bond distance between bonded atoms *i* and *j*, and *b_ij_*_,0_ is the reference bond distance. For simplicity, we adopt the parameters from the Martini 2 model, κ_*b*_ = 2000 kJ mol^–1^ nm^–2^ and *b*_*ij*,0_ = 0.46 nm,^39,40^ since the Martini 3 models employ different parameters for bonds in the chain interior and at the ends (terminal bonds).

The dihedral distribution for the mapped AA model is broad and weakly structured, so we omit an explicit dihedral potential in our model (see Sec. I of the SI). In the absence of a dihedral potential and with a large bond force constant (i.e., an approximately fixed bond length), PE can be approximated as a discrete worm-like chain (WLC), whose fluctuations are dominated by small bending angles Δθ. We consider the standard harmonic angle potential (eq 2) instead of the temperature-dependent G96 potential (eq 1) used in the Martini 3­(v2) model. The temperature dependence of the force constant in the G96 angle potential for Martini 3­(v2) is shown in Sec. III of the SI.

By setting θ_*ijk*,0_ = 180° and defining −Δθ = (θ*_ijk_* – θ*_ijk_*_,0_) in eq 2,

11 $$V_{a}\left(\Delta \theta \right)=\frac{1}{2}\;\kappa_{\theta }\;{\left(\Delta \theta \right)}^{2}$$

we have Δθ as the angle between consecutive forward bond vectors in eq 7. This formulation allows us to relate the angle force constant to the persistence length:^45^

$$\kappa_{\theta }=\frac{l_{p}}{l_{b}}\;kT=N_{p}kT\;=\frac{{\left(l_{p}l_{b}\right)}_{\mathrm{AA}}}{{\left(l_{b}^{2}\right)}_{\mathrm{CG}}}\;kT$$ 12

where the quantities in the first line are defined in eq 7, and *k* is the Boltzmann constant. Details of this relation are provided in Sec. II of the SI. The second line follows from ⟨*R* ^2^⟩ of a WLC,^44^

13 $$\left\langle R^{2}\right\rangle \approx 2\;l_{p}\;l_{b}\;N\;\cos\left(\left\langle \Delta \theta \right\rangle /2\right)$$

where *N* is the number of monomers. Using the AA model mapped every two monomers (labeled AA here), we construct the CG model so that its mean-square end-to-end distance matches that of the mapped AA model:

$$\begin{array}{c} \begin{array}{c} \langle R^{2}\rangle_{\mathrm{AA}} \end{array}=\langle R^{2}\rangle_{\mathrm{CG}}\\ \begin{array}{c} {\left(l_{p}l_{b}\right)}_{\mathrm{AA}} \end{array}={\left(l_{p}l_{b}\right)}_{\mathrm{CG}}, \end{array}$$ 14

since *N* and the cosine term cancel out.

For our CG model, (*l_b_*)_CG_ = *b_ij_*_,0_ = 0.46 nm (Table 1). The quantity (*l_p_l_b_*)_AA_ in eq 12 is obtained from AA simulations at fixed temperatures ranging from 410 to 500 K in steps of 5 K for PE50 and PE100. Applying eq 12 to either the PE50 or PE100 data set gives the same average value of κ_θ_ = 8.5 ± 0.1 kJ mol^–1^ rad^–2^. Table 1 summarizes the bonded and nonbonded potentials and their parameters for our Martini-type PE model. The mass of the C1 bead is set to 54 u (unified atomic mass unit) to reproduce the densities of the AA reference systems. We show later that our Martini-type PE model reproduces the melt properties of the AA reference and also crystallizes.

**Table 1:** Bonded and Nonbonded Interaction Potentials with Parameters for Our Martini-Type PE Model ^a^

Bonded Interactions	
Interaction	Parameters
Bond Stretching (eq 10)	κ_*b*_ = 2000 kJ mol^–1^ nm^–2^; *b*_*ij*,0_ = 0.46 nm
Bond Angle (eq 2)	κ_θ_ = 8.5 kJ mol^–1^ rad^–2^; θ_*ijk*,0_ = 180°

Nonbonded Interactions	
Interaction	Parameters
12–6 LJ potential ^b^ (eq 3)	σ = 0.47 nm; ε = 3.5 kJ/mol

a We use the apolar C1 bead type with a mass of 54 u (unified atomic mass unit) to reproduce the densities of the AA models.

b Bonded beads are excluded from LJ interactions, following standard Martini practice.^22^

## 3 Results and Discussion

### 3.1 Melt Properties

Our Martini-type PE model accurately reproduces the conformational properties of PE melts from AA simulations for varying degrees of polymerization. Figure 2 compares these properties at 450 K. Both our model and Martini 3­(v2) closely match the AA reference root-mean-square radius of gyration and end-to-end distance, whereas deviations in other available models grow as the number of CG beads increases. For the density, the Martini 3 models use a bead mass of 72 u. To compare with our model and Martini 2 (54 u), we scale the Martini 3 densities by (54/72) in Figure 2, which is justified for these homogeneous PE melts. Deviations are around 4% for short chains (10 CG beads) in both our model and Martini 2, decreasing to a plateau of approximately 1.5% for longer chains. Because the total number of chains is the same across all degrees of polymerization in our simulation design (Secs 2.1 and 2.2), density fluctuations decrease for longer chains due to the increasing number of interior segments and statistical averaging over more particles.

**Figure 2:**
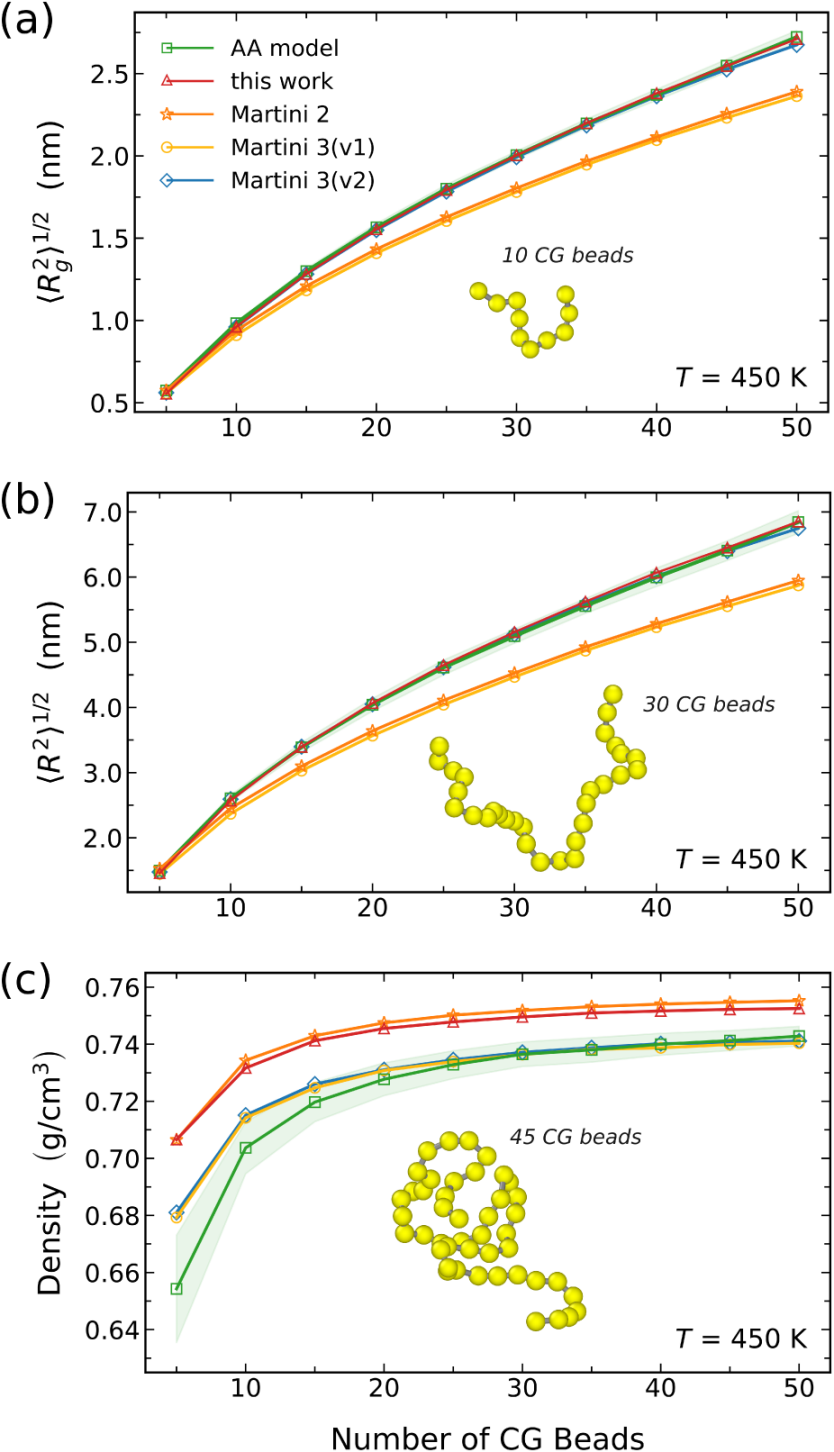
Polymer chain statistics at 450 K as a function of the number of CG beads for our Martini-type polyethylene (PE) model, available Martini PE models, and the mapped AA reference. (a) Root-mean-square (RMS) radius of gyration (eq 5), (b) RMS end-to-end distance (eq 4), and (c) density. Shaded region indicates standard deviation of the AA model; standard deviations of the coarse-grained PE models are similar and omitted for clarity. Single-chain snapshots of our model: apolar C1 beads (yellow) and bonds (gray rods).

Our Martini-type model reproduces the temperature-dependent conformational properties of melt PE50 from AA simulations (Figure 3). Both our model and Martini 3­(v2) accurately reproduce the RMS radius of gyration and RMS end-to-end distance of the AA reference. This agreement is expected because the angle force constant in our model is parametrized using the end-to-end distance of the AA model (Sec 2.4). For ideal chains, such as those in a PE melt, the end-to-end distance and radius of gyration are related by $\left\langle R^{2}\right\rangle =6\left\langle R_{g}^{2}\right\rangle$ ,^44,45^ so targeting one naturally reproduces the other. The angle force constant κ_θ,G96_ in Martini 3­(v2) should vary with temperature to match our force constant κ_θ_ in Table 1. Over the temperature range shown in Figure 3, the converted κ_θ,G96_ values remain close to the fixed choice κ_θ,G96_ = 20 kJ/mol used in Martini 3­(v2), with deviations that increase as the temperature decreases. This explains the similar trends observed for our model and Martini 3­(v2) in Figures 2a,b and 3a,b. Section III of the SI shows the converted κ_θ,G96_ values obtained from our angle force constant.

**Figure 3:**
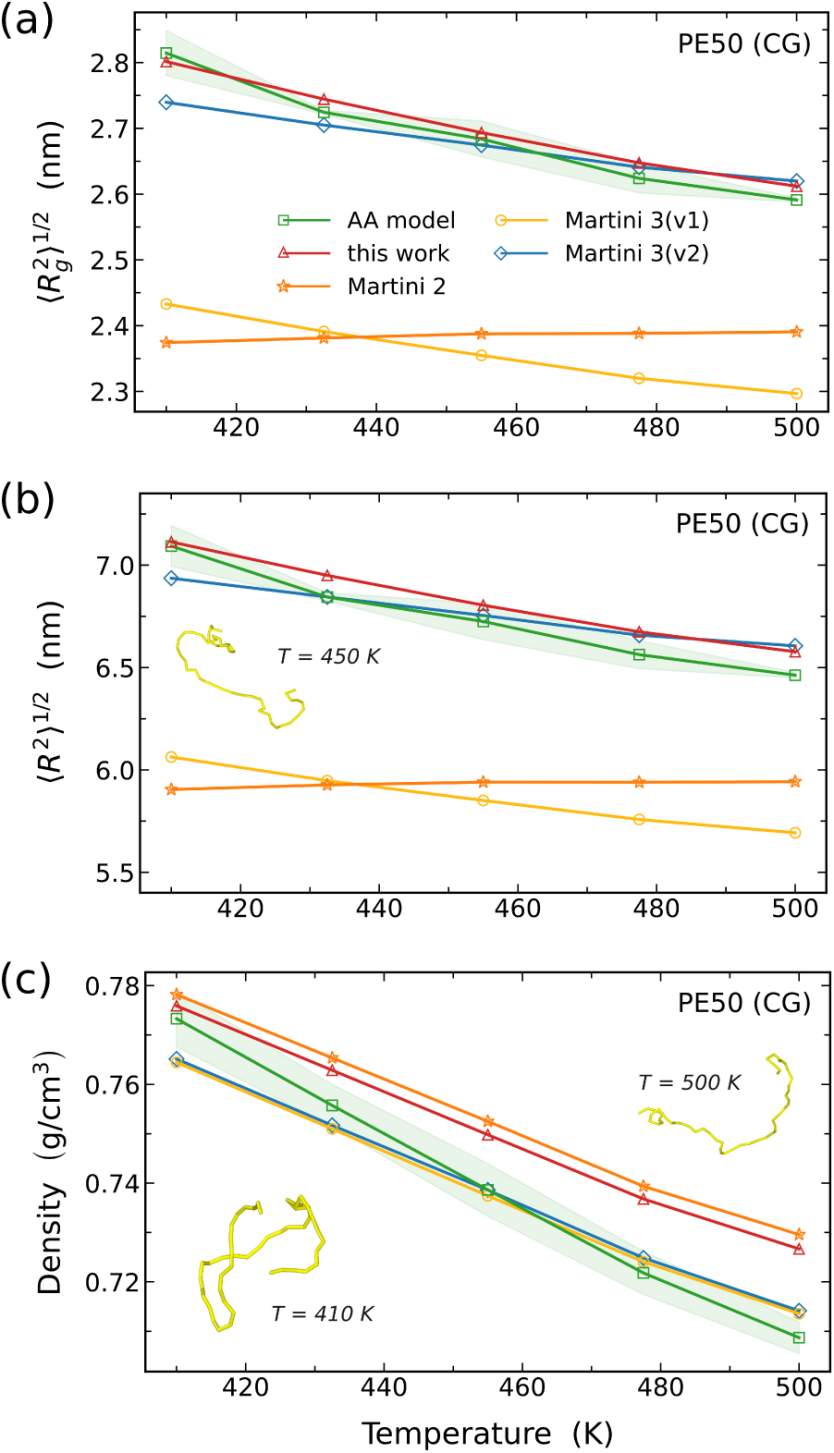
Polymer chain statistics for PE100 (PE50 (CG)) as a function of temperature for our Martini-type polyethylene (PE) model, available Martini PE models, and the mapped AA reference. Panels show (a) RMS radius of gyration (eq 5), (b) RMS end-to-end distance (eq 4), and (c) density. Shaded region indicates standard deviation of the AA model; standard deviations of the coarse-grained PE models are similar and omitted for clarity. Single-chain snapshots of our model at different temperatures using a line representation (yellow).

Martini 2 shows a contrasting trend for both the end-to-end distance and radius of gyration with temperature (Figure 3a,b). For a constant κ_θ_ and fixed *l*_*b*_ in eq 12, the persistence length decreases as temperature rises, so both the end-to-end distance and radius of gyration are expected to follow the same trend (eq 13). This behavior has also been observed experimentally.^48^ All models except Martini 2 follow this trend (Figure 3). Experimentally, the RMS radius of gyration obeys $\langle R_{g}^{2}\rangle^{1/2}=\left(0.046\pm 0.005\right)MM^{1/2}$ , where *MM* is the molar mass in g/mol and $\langle R_{g}^{2}\rangle^{1/2}$ is in nanometers.^49^ This relation has been tested for short (MM = 1400) and long (MM = 81000) chains,^50^ but the experimental uncertainty prevents a temperature-dependent analysis. The RMS radius of gyration of the AA reference lies within this range. The density from our model deviates by less than 1% at 410 K and by about 2.7% at 500 K relative to the AA model (Figure 3c). Statistical properties for PE25 (CG) as a function of temperature are provided in Sec. IV of the SI and are equivalent to those for PE50 (CG). The CG models capture the melt structure of the AA reference (Sec. V of the SI), and differences between the unmapped and mapped AA models are negligible, as the AA model is mapped every two monomers (see Sec. VI of the SI).

### 3.2 Crystal Properties

Polyethylene chains modeled with Martini 2 and Martini 3 (v2) do not crystallize within accessible simulation times. Even in longer simulations (3 μs) over 250–500 K in 5 K increments, no nucleation is observed. Martini 3­(v1) exhibits crystallization at 255 K; however, the crystals fully melt at body temperature (310 K), inconsistent with the expected semicrystalline structure. In contrast, our Martini-type PE model crystallizes. We compare its crystalline structure with the AA reference in Figure 4. Both form hexagonal structures, with lattice parameters *a* = 0.53 ± 0.01 nm (our model) and 0.49 ± 0.01 nm (AA), in good agreement with the experimental value of 0.488 nm.^51^ Although hexagonal structures typically occur at high pressures, they are also observed in small crystallites at lower pressures.^52^ The interchain radial distribution function (eq 9) in Figure 4 reproduces the projection of a hexagonal lattice along the [001] direction where the PE backbones align with the *z*-axis. A basis for this projection can be defined as^53^

$$\begin{array}{c} \begin{array}{c} a_{1} \end{array}=a\left(1,0\right)\\ a_{2}=a\left(-\frac{1}{2},\frac{\sqrt{3}}{2}\right), \end{array}$$ 15

and the vector connecting two lattice points is

16 $$R_{mn}=m\;a_{1}+n\;a_{2}$$

where *m* and *n* are integers. The distance between two lattice points is given by the norm of eq 16:

17 $$R_{mn}=a\sqrt{m^{2}-mn+n^{2}}$$

corresponding to discrete coordination shells. At 350 K, thermal broadening smooths these shells, so the interchain RDF in Figure 4 exhibits transitions between successive shell distances $R_{mn}/a=r/a=1,\;\sqrt{3},\;2,\;\sqrt{7},\;3,\;2\sqrt{3},\;...$ , rather than sharp peaks at each value. We estimate the melting temperatures of these hexagonal structures in Sec. VII of the SI.

**Figure 4:**
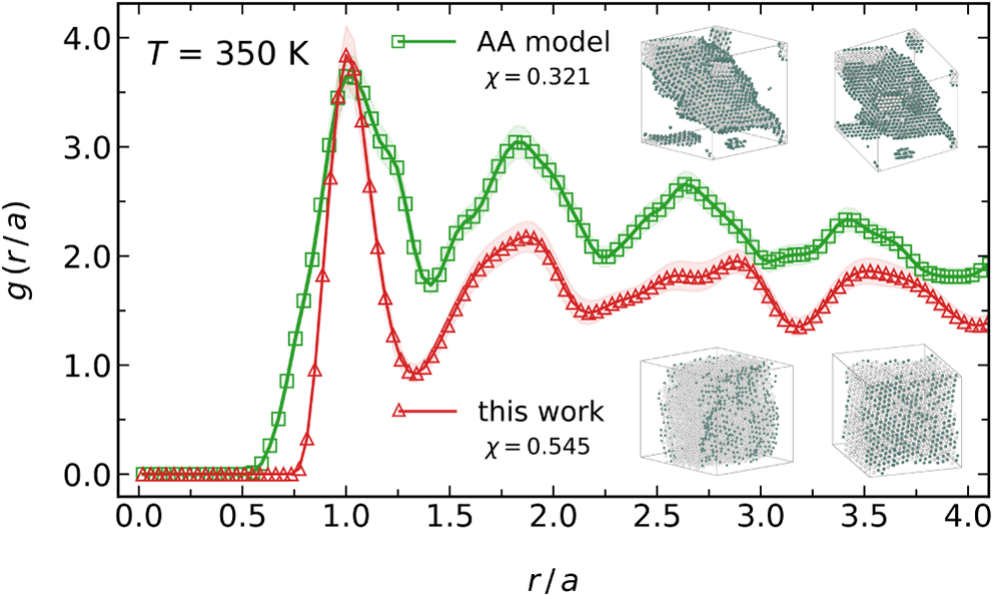
Interchain radial distribution function (RDF) for PE100 (PE50 (CG)) at 350 K. Shaded regions indicate standard deviations. Snapshots adjacent to each legend: crystalline atoms (white and dark green) and crystallinity χ associated with the AA model and our PE model. The unmapped and mapped AA models yield identical results.

### 3.3 Nanoplastic–Membrane Interactions

We generate nanoplastics in water using our PE model, consisting of chains with 50 CG beads and two different chain counts (25 and 50), following the procedure in refs 11, 31 with one modification. Instead of annealing from a higher temperature to body temperature (310 K), we first equilibrate the nanoplastic-water system at 280 K for 250 ns to accelerate nucleation, using the parameters in Sec 2.2.The system is then heated at a rate of 3 K/ns using the postequilibration parameters from the same section, followed by an additional 90 ns run at 310 K. Section VIII of the SI provides an analysis of the NPL structures under this setup. The water is modeled using the standard Martini nonpolarizable bead representing four water molecules.

Nanoplastics generated with our Martini-type PE model exhibit semicrystalline morphology consistent with the AA reference. Representative nanoplastics for each chain count are shown in Figure 5a,b, together with comparisons to the AA reference and the Martini 2 model, which also represents other Martini PE models lacking a crystalline phase at 310 K. The NPLs for the AA and Martini 2 models are taken from ref ^11^. Our Martini-type PE model reproduces the expected semicrystalline structure of the AA model. Table 2 summarizes the properties of the nanoplastics shown in Figure 5a,b. We triangulate the nanoplastic surface with a convex hull algorithm^54^ applied to the carbon atoms (CG beads) to determine the equivalent spherical radius *R* that reproduces the surface area. The radius of gyration is computed from the center of mass of the nanoplastic. Deviations from a spherical shape are quantified by the ratio *R*/*R*_*g*_, which equals $\sqrt{5/3}$ for a perfect sphere.^44^ These deviations are larger for nanoplastics with 25 chains, especially for models with crystalline domains. Because crystallization is stochastic, nanoplastics may exhibit varying degrees of crystallinity.^31^ As shown in Sec 3.4, the AA model can further crystallize once embedded in the lipid membrane, reaching a crystallinity comparable to that of our PE model.

**Figure 5:**
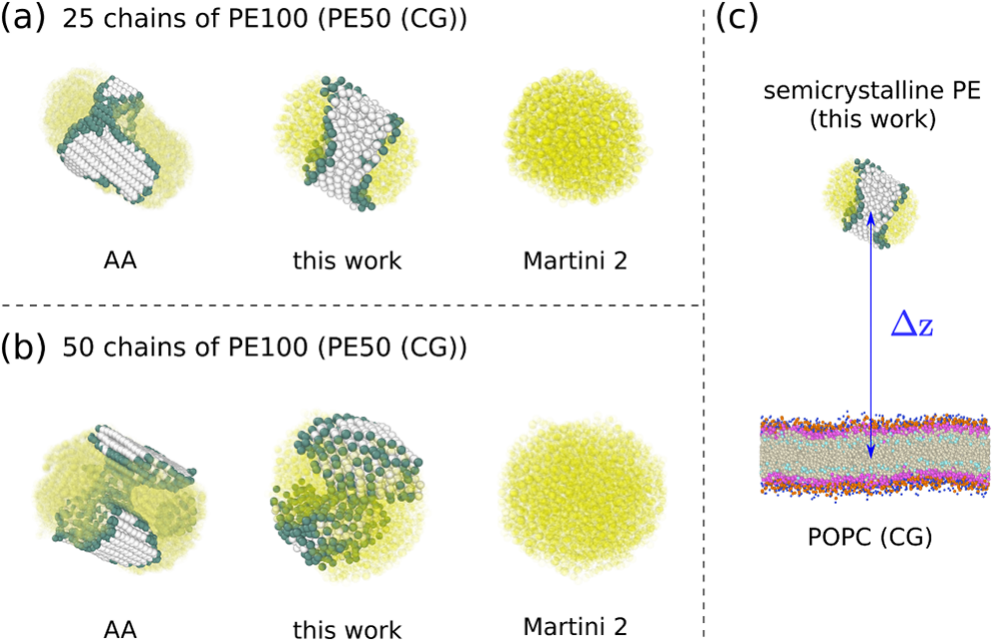
Comparison of nanoplastics formed from PE100 (PE50 (CG)) across different models and chain counts at 310 K (body temperature) and 1 bar. Panels show (a) 25 chains and (b) 50 chains. Crystalline atoms (white and dark green). (c) Reaction coordinate Δ*z*, the distance between the center of mass of the NPL and the lipid membrane, used to compute the potential of mean force (PMF). Nanoplastics from the Martini 3 models are similar to those from Martini 2, as the crystalline phase is absent.

**Table 2:** Properties of Nanoplastics Shown in Figure 5a,b: Equivalent Spherical Radius *R*, Radius of Gyration *R_g_*, Deviation from Spherical Shape, and NPL Crystallinity χ

Nanoplastics	*R* (nm) ^a^	*R*_*g*_ (nm) ^b^	Sphericity Deviation (%) ^c^	NPL Crystallinity χ ^d^
PE50 (AA)^11^	3.98	3.11	0.87	0.280
PE25 (AA)^11^	3.20	2.62	5.39	0.281
PE50 (this work)	3.90	3.09	2.24	0.383
PE25 (this work)	3.09	2.51	4.64	0.456
PE50 (Martini 2)^11^	3.98	3.10	0.55	0
PE25 (Martini 2)^11^	3.11	2.46	2.07	0

a Equivalent spherical radius *R* that yields the same NPL surface area.

b Radius of gyration computed with respect to the NPL center of mass using all polymer chains.

c Deviation from a perfect sphere, for which $R/R_{g}=\sqrt{5/3}$ .^44^

d Defined in Sec 2.3.4.

With the two semicrystalline nanoplastics from our Martini-type model in Figure 5a,b, we compute the potential of mean force (PMF) between these NPLs and a POPC membrane described by the Martini 2 force field.^55^ The POPC membrane is constructed using the *Martini Maker* module in CHARMM-GUI^34,56^ and contains the same number of lipids in each leaflet as in ref 11 for comparison. Figure 5c illustrates the reaction coordinate, where the NPL is first pulled toward the membrane to generate a series of configurations. The PMF is then computed using the Weighted Histogram Analysis Method (WHAM)^57^ combined with the bootstrap technique, employing the same parameters as in ref 11. The PMF is referenced to zero when the NPL enters the membrane.

To generate configurations for umbrella sampling and extract the PMF, we perform a steered MD simulation for 100 ns using an umbrella potential with a harmonic force constant of 2000 kJ mol^–1^ nm^–2^. The generated configurations are equilibrated at 310 K and 1 bar for 5 ns under the NPT protocol described in Sec 2.1, while restraining the NPL position with the same umbrella potential. Umbrella sampling simulations are then performed for 100 ns using the same force constant, NPT conditions, and postequilibration protocols (Sec 2.1). The PMF is obtained by averaging 150 bootstrap PMFs with a WHAM convergence tolerance of 1.0 × 10^–6^.

The POPC membrane exhibits unphysical bending toward our semicrystalline NPLs and mixes with them. For the NPL with 25 chains (Figure 6a), the PMF shows a barrier of approximately 46*kT*, which is overcome as the NPL enters the membrane and becomes attracted to the hydrophobic core. Oscillations along the reaction coordinate correspond to configurations in which the membrane bends toward the NPL and partially mixes with it. This unphysical behavior contrasts with the purely repulsive PMF profiles obtained from AA references.^11^ For the NPL with 50 chains (Figure 6b), no free-energy barrier is observed, and the NPL penetrates the membrane without a free-energy cost. This occurs because the same unphysical membrane deformation observed for the NPL with 25 chains is now present during the pulling process. The membrane’s tendency to bend toward and mix with the NPL is reflected in a shift of the zero reference toward the NPL in Figure 6b. The PMF curves for both NPLs become similar once their zero references are aligned (Figure 6c).

**Figure 6:**
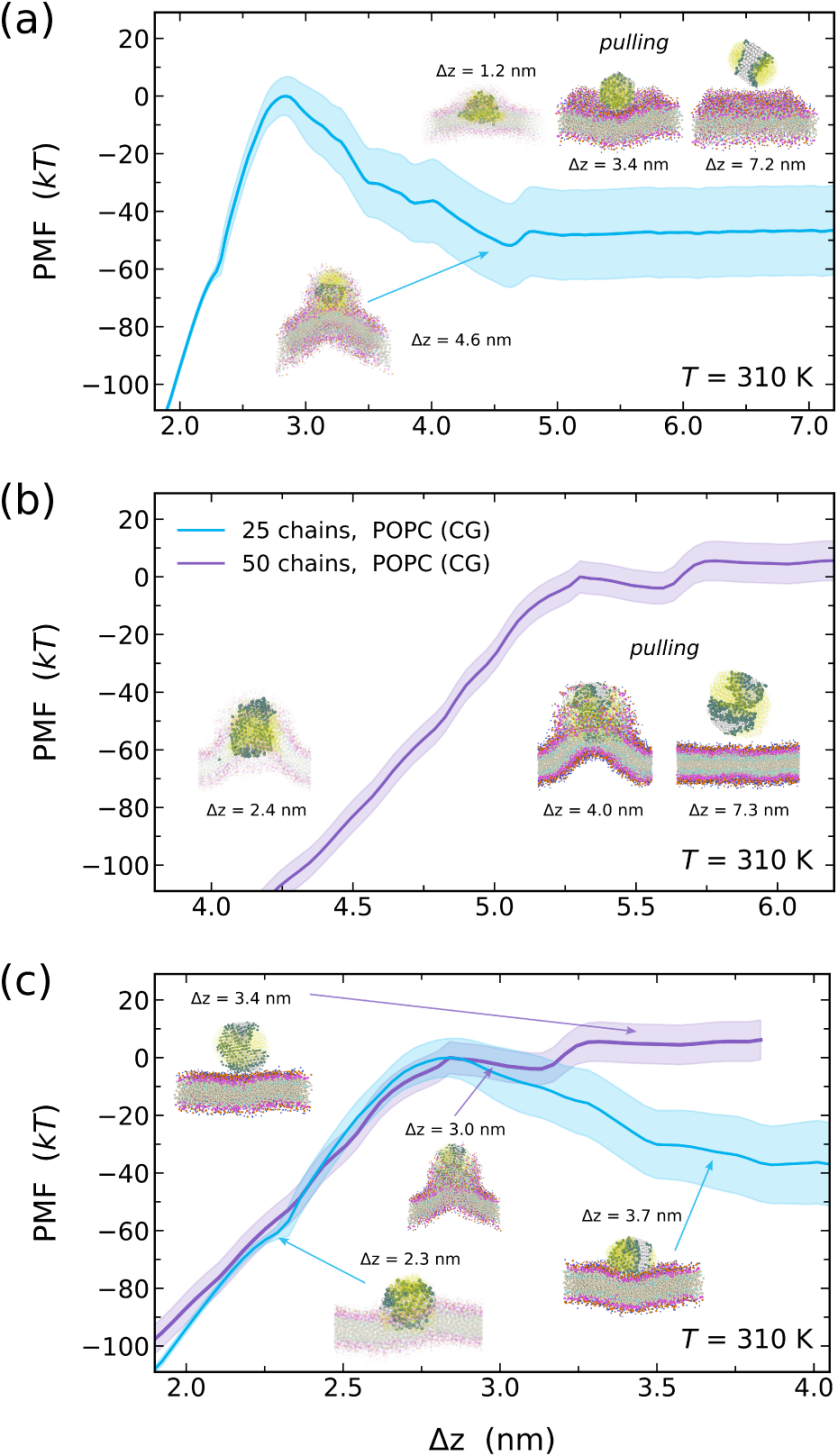
Potential of mean force (PMF) between a POPC membrane and two nanoplastics using our Martini-type PE model. (a) POPC (CG)–NPL with 25 chains (Figure 5a) and (b) POPC (CG)–NPL with 50 chains (Figure 5b). The zero of the PMF is defined when the NPL translocates into the membrane interior. (c) The PMF curve in (b) is shifted by 2.46 nm to align its reference with that in (a). Shaded regions indicate standard deviations. Snapshots: pulling process along Δ*z*; colored arrows indicate representative configurations during the PMF calculations.

### 3.4 Nanoplastic Embedded within the Membrane Core

To explore the behavior of an NPL embedded within the membrane core, we place the NPL from our Martini-type PE model with 25 chains (Figure 5a) inside a POPC (CG) membrane surrounded by water along the *z*-axis (Figure 7c). The system is first equilibrated at 310 K and 1 bar for 1 ns, allowing the NPL to accommodate within the membrane core before data collection. The NPL dynamics are then monitored for 600 ns and compared to the AA model in Figure 7. Equilibration and postequilibration parameters are provided in Sec 2.2, and the AA model data are taken from ref 11.

**Figure 7:**
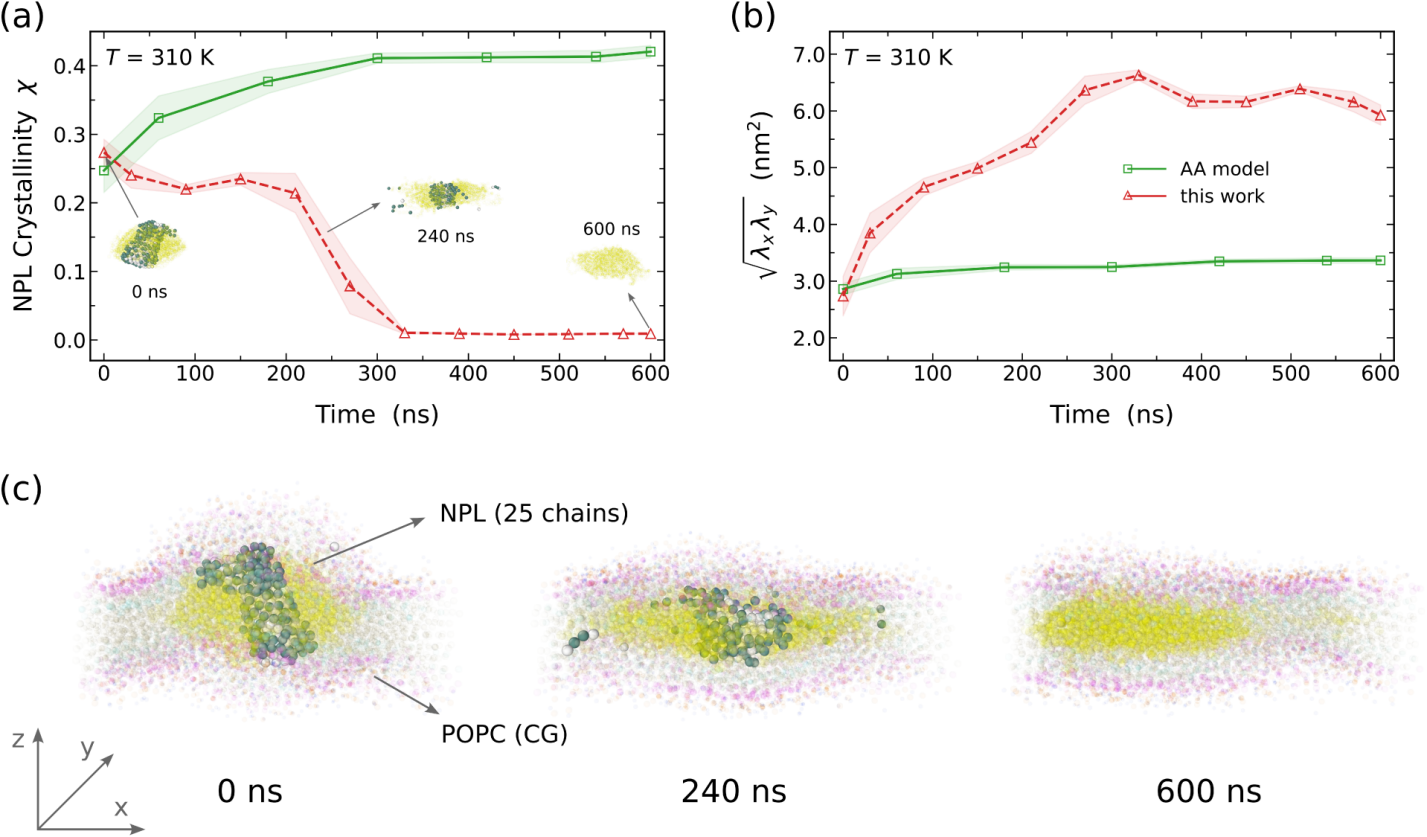
Nanoplastic embedded within the membrane core. (a) NPL crystallinity χ and snapshots of our PE model. (b) Lateral spread of the NPL $\sqrt{\lambda_{x}\lambda_{y}}$ . (c) Snapshots of our semicrystalline PE model within the POPC membrane core (Martini 2): crystalline atoms (white and dark green); water omitted for clarity. Shaded regions indicate standard deviations. Data for the AA model are taken from ref^11^.

While the semicrystalline NPL in the AA model remains intact with no lateral spread, our coarse-grained semicrystalline NPL spreads laterally within the membrane core (Figure 7b). This spreading reflects dissolution of our PE model within the lipid environment, leading to loss of crystallinity (Figure 7a). In the AA model, hydrophobic lipid tails further promote NPL crystallization, increasing the NPL crystallinity χ, which can be compared to the crystallinity of our coarse-grained model in Table 2. Lateral spread of the NPL is quantified by $\sqrt{\lambda_{x}\lambda_{y}}$ , where λ_*x*_ and λ_*y*_ are the eigenvalues of the NPL mass gyration tensor (eq 6) corresponding to the *xy*-plane.

## 4 Conclusion

We present an improved Martini-type model for polyethylene (PE). This model is compared with three current Martini PE models from the Martini 2 and Martini 3 force fields, and their melt and crystal properties are benchmarked against all-atom (AA) simulations and experimental data. The current Martini models reproduce PE melt properties well, as their development primarily targeted these properties. Our model also captures the melt properties of the AA reference, performing on par with the best-performing Martini 3­(v2) model. For crystal properties, our model reproduces the semicrystalline morphologies, crystal structure, and lattice parameters of the AA model, whereas the current Martini models either do not crystallize (Martini 2 and Martini 3­(v2)) or do not form the expected crystalline domains at physiological temperatures (Martini 3­(v1)), such as 310 K (body temperature).

Nanoplastics generated from our PE model are comparable to those from the AA reference. We use our PE model to construct semicrystalline NPLs similar to those in the AA model and benchmark their properties, including the radius of gyration and crystallinity. In contrast, PE NPLs from current Martini models remain amorphous at 310 K, which is inconsistent with AA simulations and experimental observations.

The interaction between our semicrystalline NPLs and the Martini POPC lipid membrane exhibits the same unphysical behavior reported previously for amorphous NPLs. The lipid membrane bends toward the nanoplastic and mixes with it. Placing a semicrystalline NPL within the POPC (CG) membrane also leads to behavior that deviates from the AA reference. While the AA model shows that the NPL remains intact and that the lipid environment promotes further crystallization, our Martini-type model spreads laterally within the membrane and dissolves in the lipid “solvent”, losing its crystalline domains. These contrasting behaviors relative to the AA reference indicate that improving the PE model alone is insufficient to accurately characterize alkane–membrane interactions, and that refinement of the membrane model is also necessary. A first step toward this goal would be to adjust the cross LJ interactions between PE and membrane alkyl beads. The incompatibility between the two alkyl components can be quantified by an effective mixing-energy mismatch $\Delta \varepsilon_{mix}∼2\varepsilon_{PM}-\varepsilon_{M}-\varepsilon_{P}$ (motivated by the exchange process: *M*–*M* + *P*–*P* → 2 *M*–*P*), where ε_*PM*_ is the cross LJ interaction strength and ε_*P*_ and ε_*M*_ are the LJ parameters for the polymer (*P*) and membrane (*M*) alkyl beads, respectively. Tuning ε_*PM*_ could therefore be used to adjust PE–lipid mixing behavior. However, we leave this task for future work.

Overall, we expect this work to advance the understanding of alkane–membrane interactions and to inform strategies for improving the Martini force field. It also paves the way for reliable coarse-grained models of nanoplastics in large biological systems that are currently beyond the reach of all-atom simulations.

## Supplementary Material



## Notes

Additional details of the studied systems; distributions of bonded interactions; temperature dependence of the G96 angle potential; polymer chain statistics for PE25 (CG); interchain radial distribution function of PE melts; comparison between unmapped and mapped AA models­(PDF)

## †

11100 Johns Hopkins Road, Johns Hopkins Applied Physics Laboratory, Laurel, Maryland 20723, United States
