# Supplementary material for: Assessing the Martini Force Field for Modeling Polyolefin Nanoplastics near Lipid Membranes
Source: J Phys Chem B. 2026 Jun 15;130(28):7145–54. doi: 10.1021/acs.jpcb.6c02507 (PMC13383732; doi:10.1021/acs.jpcb.6c02507)
Supplement: Supplementary file 1 [file jp6c02507_si_001.pdf]

Supplementary Information for "Assessing the Martini Force Field for Modeling Polyolefin Nanoplastics near Lipid Membranes"

Anderson D. S. Duraes,[†] Caleb Liu,[†,‡] and Wenlin Zhang[*,†]

†41 College Street, Department of Chemistry,

Dartmouth College, Hanover, New Hampshire 03755, United States

‡Current address: 11100 Johns Hopkins Road,

Johns Hopkins Applied Physics Laboratory, Laurel, Maryland 20723, United States

E-mail: wenlin.zhang@dartmouth.edu

Contents

I Distributions of Bonded Interactions S3

II Persistence Length S7

III Temperature Dependence of the G96 Angle Potential S8

IV Polymer Chain Statistics for PE25 (CG) S11

V Interchain Radial Distribution Function of PE Melts S12

VI Comparison Between Mapped and Unmapped AA Models S13

VII Melting and Nucleation Behavior of the Martini-Type PE Model S14

VIII Nanoplastic Stability S15

References S16

I Distributions of Bonded Interactions

The bonded distributions for polyethylene at 450 K are shown in Fig. S2 and are independent of chain length, as the chains share a common mapping scheme. The bond-stretching distribution in the AA model exhibits two peaks that arise from mapping two monomers to one bead. Without loss of generality, if the first carbon is taken as the mapping position, this bond distance corresponds to the separation between the first and fifth carbons in the AA model (Fig. S1), involving a five-carbon segment and corresponding to a sequence of two rotational isomeric states (RIS). We adopt the RIS notation and angle ranges reported in Table 2 of Ref. 1. The peak at approximately 0.46 nm is associated with the TG, Tg, their reverse states, and XX. The peak at approximately 0.51 nm corresponds to the TT state, while the region between these peaks corresponds to the TX and XT states. For clarity, Table S1 summarizes the dihedral pair states, their average bond distances, and relative populations. The bond-stretching distribution for the Martini models, however, shows a single maximum, which smears out the conformational states observed in the AA model, since no internal conformations exist between two C1 beads.

The angle distribution for the model developed in this work and for Martini 3(v1) better reproduces the distribution of the AA model. Because the target dihedral distribution from the AA model is broad and weakly structured, no dihedral potential is included in our coarse-grained model. The Martini 3(v2) model follows the same approach and therefore exhibits a nearly uniform dihedral distribution over the entire dihedral range (see Sec. II.2 of the main paper).

We compare different AA-to-CG mappings in Fig. S3. Taking the CG bead position as either the first (C(1)) or second (C(2)) carbon atom of the two-monomer unit does not change the statistical properties of the mapped AA model, leading to nearly identical bond, angle, and dihedral distributions. In contrast, defining the CG bead position as the center of mass (COM) of each two-monomer unit averages over intra-segment torsional degrees of freedom. This smears out the dihedral states and systematically renormalizes the bonded distributions.

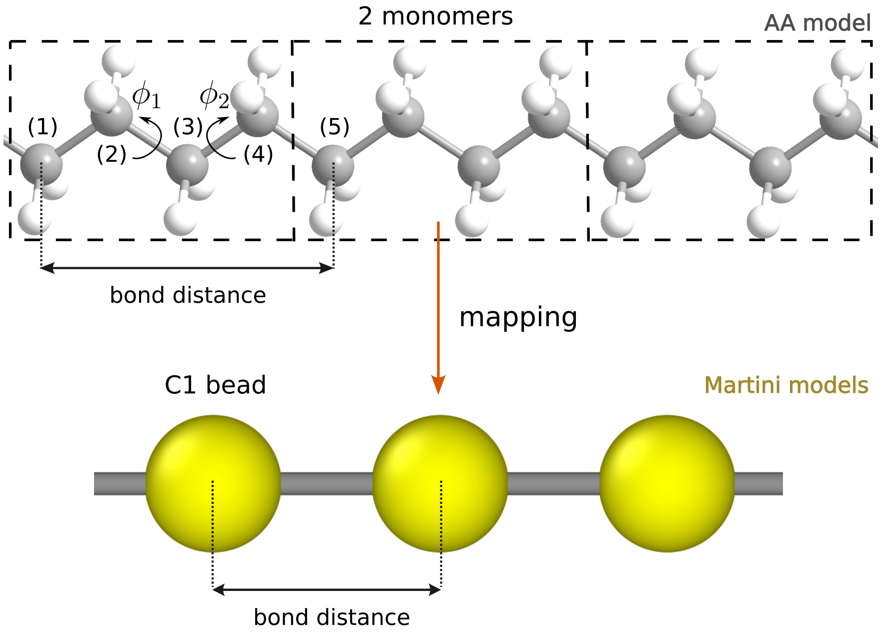

Figure S1: Bond distance in the all-atom (AA) model (reference) involves five carbon atoms (numbered) and depends on the conformation of the dihedral pair (ϕ_1, ϕ_2) (see Table S1). In the Martini models, the bond distance involves only two C1 beads and is therefore independent of dihedral conformations.

Table S1: Rotational isomeric state (RIS) pairs identified from the conformations of five consecutive carbons in the AA model (mapping every two monomers; see Fig. S1), along with their corresponding average bond distances and relative populations at 450 K.

State Pair [a]	Average Bond Distance (nm)	Relative Population (%) [b]
TT	0.511	32.35
TX, XT	0.491	12.73
XX	0.469	1.03
TG, GT, Tg, gT	0.461	40.44
GX, gX, XG, Xg	0.435	7.06
GG, gg	0.395	5.94
Gg, gG	0.344	0.45

[a] We adopt the RIS notation and angle ranges reported in Table 2 of Ref. 1.

[b] The relative population is the sum of the contributions from each state pair. States listed within the same row contribute equally; for example, the states TG, GT, Tg and gT are statistically equivalent.

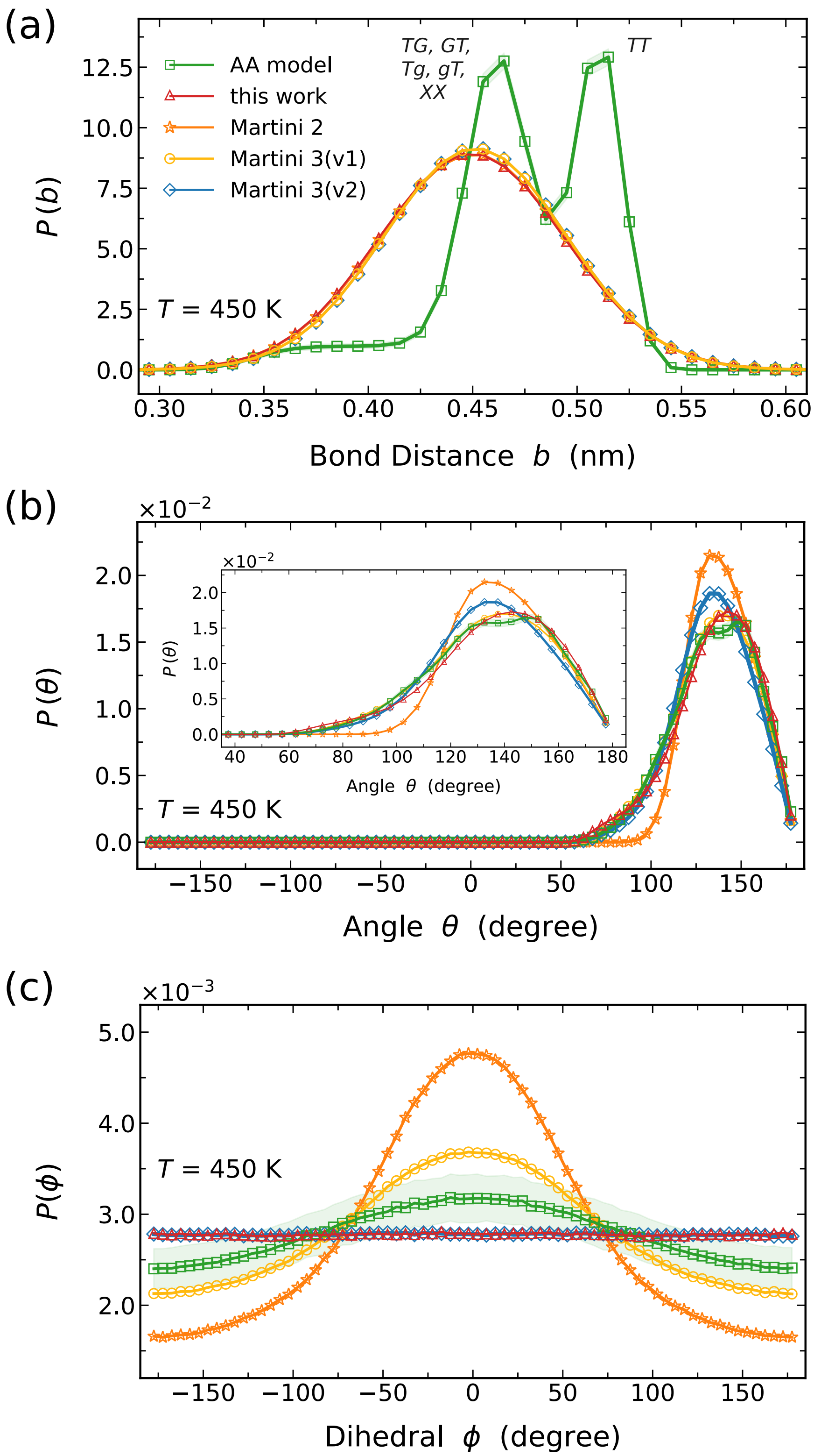

Figure S2: Bonded distributions (probability density) for polyethylene (PE) with 50 C1 beads (100 monomers) at 450 K: (a) bond stretching, (b) bond angle, and (c) dihedral angle distributions for the all-atom (AA) model (reference) and the Martini PE models. The states in (a) correspond to the two green peaks (see Table S1). Shaded region indicates standard deviation.

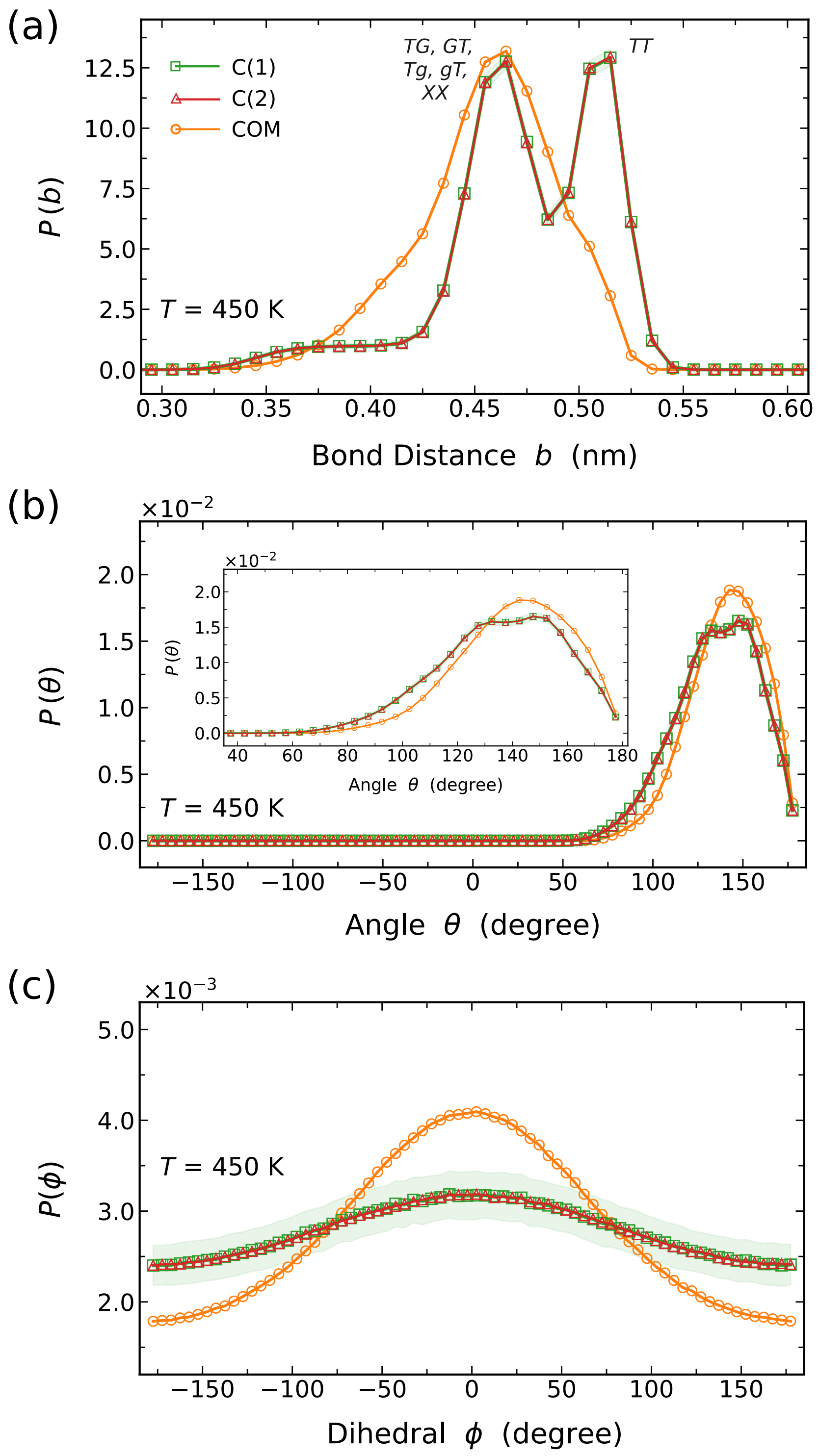

Figure S3: Comparison of bonded distributions (probability density) for polyethylene (PE) with 50 CG beads (100 monomers) at 450 K using different AA-to-CG mappings: C(1) uses the first carbon atom of each two-monomer unit, C(2) uses the second carbon atom, and COM uses the center of mass. (a) Bond stretching, (b) bond angle, and (c) dihedral angle distributions. The states in (a) correspond to the overlapping green/red peaks (see Table S1). Shaded region indicates standard deviation.

II Persistence Length

We compute the orientational correlations (Eq. (7) in the main text) for the worm-like chain model as:[2]

$$\begin{aligned}\langle \hat{\mathbf{b}}_s(0)\cdot\hat{\mathbf{b}}_s(n)\rangle_s &= [\langle \cos\Delta\theta\rangle_s]^n \\ &\approx \left[1-\frac{\langle\Delta\theta^2\rangle_s}{2}\right]^n = 1-\frac{n\,\langle\Delta\theta^2\rangle_s}{2} \\ &\approx \exp\left(\frac{-n\,\langle\Delta\theta^2\rangle_s}{2}\right)\,,\end{aligned} \tag{S1}$$

where we use the small-angle approximations for $(\cos x)$, $(1-x)^n$ and $(\exp x)$, and assume that successive bond orientations are statistically independent and identically distributed [3, 4]

$$\begin{aligned}\langle \hat{\mathbf{b}}_s(0)\cdot\hat{\mathbf{b}}_s(n)\rangle_s &= \langle \cos\Delta\theta_{0,1}\,\cos\Delta\theta_{1,2}\,\cdots\,\cos\Delta\theta_{n-1,n}\rangle_s \\ &= \langle\cos\Delta\theta_{0,1}\rangle_s\,\langle\cos\Delta\theta_{1,2}\rangle_s\,\cdots\,\langle\cos\Delta\theta_{n-1,n}\rangle_s \\ &= [\langle\cos\Delta\theta\rangle_s]^n\end{aligned} \tag{S2}$$

where $\Delta\theta_{a,b} = \Delta\theta$ denotes the angle between successive bond vectors with indices a and b, which are assumed to be identically distributed. From Eq. (11) in the main paper, we compute the ensemble average

$$\begin{aligned}\langle\Delta\theta^2\rangle_s &= \frac{\int_0^{2\pi} d\phi\,\int_0^{\pi} d\Delta\theta\,\Delta\theta^2\,\sin\Delta\theta\,\exp(-\beta\,V_a(\Delta\theta))}{\int_0^{2\pi} d\phi\,\int_0^{\pi} d\Delta\theta\,\sin\Delta\theta\,\exp(-\beta\,V_a(\Delta\theta))} \\ &\approx \frac{2}{\beta\,\kappa_\theta}\end{aligned} \tag{S3}$$

with $\beta = (kT)^{-1}$ and using $\sin\Delta\theta \approx \Delta\theta$ for small $\Delta\theta$. Substituting Eq. (S3) into Eq. (S1) and comparing the exponential argument with that in Eq. (7) in the main paper yields the relation between the angle force constant and the persistence length:

$$\kappa_\theta = \frac{l_p}{l_b}\,kT = N_p\,kT\,. \tag{S4}$$

III Temperature Dependence of the G96 Angle Potential

We convert the angle force constants between the G96 and harmonic (h) angle potentials (Eqs. (1) and (2) in the main paper, respectively). To ensure consistent conformational statistics between the two potentials, we impose

$$\langle (\theta - \theta_0)^2 \rangle_{G96} = \langle (\theta - \theta_0)^2 \rangle_h \,, \tag{S5}$$

where we abbreviate the notation as $\theta = \theta_{ijk}$ and $\theta_0 = \theta_{ijk,0}$ for simplicity. The corresponding fluctuations around θ_0 are computed as:

$$\begin{aligned} \langle (\theta - \theta_0)^2 \rangle_m &= \frac{\int_0^{2\pi} d\phi \int_0^{\pi} d\theta \; (\theta - \theta_0)^2 \, \sin\theta \, \exp(-\beta V_m)}{\int_0^{2\pi} d\phi \int_0^{\pi} d\theta \, \sin\theta \, \exp(-\beta V_m)} \\ &= \frac{\int_0^{\pi} d\theta \; (\theta - \theta_0)^2 \, \sin\theta \, \exp(-\beta V_m)}{\int_0^{\pi} d\theta \, \sin\theta \, \exp(-\beta V_m)} \,, \end{aligned} \tag{S6}$$

where $m = \text{G96}$ corresponds to the G96 angle potential $V_m = V_{a,G96}(\theta)$ (Eq. (1) in the main paper) and $m = h$ corresponds to the harmonic angle potential $V_m = V_a(\theta)$ (Eq. (2) in the main paper). For a given reference value θ_0 and one of the angle force constants, we determine the other force constant by searching for the value that minimizes the squared difference between the two sides of Eq. (S5) using the Powell algorithm. [5] The integrals in Eq. (S6) are evaluated numerically. [6]

The GROMOS manual [7] provides a different approach to converting κ_θ to $\kappa_{\theta,G96}$. By equating the two angle potentials and invoking the equipartition theorem, the manual reports:

$$\kappa_{\theta,G96} = \frac{2kT}{[\cos(\theta_0 + \delta) - \cos\theta_0]^2 + [\cos(\theta_0 - \delta) - \cos\theta_0]^2} \,, \tag{S7}$$

where $\delta = \sqrt{kT/\kappa_\theta}$.

The GROMACS manual [8] also provides an alternative conversion:

$$\left(\kappa_{\theta,G96}\right)\,\sin^2\theta_0 \;=\; \kappa_\theta\;, \tag{S8}$$

which is obtained by expanding the cosine term in Eq. (1) of the main paper to first order around θ_0 and equating it to the harmonic potential. However, this relation does not apply when $\theta_0 = 180°$, where a second-order expansion of the cosine is required.

Figure S4(a) compares the conversion methods in Eqs. (S5) and (S7), mapping the harmonic angle potential in our Martini-type PE model (this work) to the G96 angle force constant. The GROMOS method yields significantly higher values than our variance-based approach. To validate the two methods, we computed polymer chain statistics of PE50 (CG) at 450 K using the converted parameters, following the same equilibration and post-equilibration protocol described in Sec. II of the main paper, and replacing only the harmonic angle potential with the G96 form. The results are reported in Table S2. The variance-based approach reproduces the polymer statistics of the target harmonic angle potential, while the GROMOS method shows large deviations, indicating a mismatch in conformational fluctuations.

The good agreement of Martini 3(v2) for melt properties arises from the fact that its angle force constant is close to our harmonic constant upon conversion. At 450 K, our κ_θ corresponds to $\kappa_{\theta,G96} = 20.645$ kJ/mol (Table S2), close to the 20 kJ/mol value used in Martini 3(v2). However, as temperature decreases, the deviation from our harmonic constant increases (Fig. S4(b)), and the polymer statistics also deviate (Fig. S4(d)). This temperature dependence of the G96 angle force constant hinders crystallization in the Martini 3(v2) model, since lower temperature implies a smaller equivalent harmonic force constant.

Table S2: Comparison of conversion methods (Eqs. (S5) and (S7)) for the harmonic angle potential in our Martini-type PE model (Table 1 in the main paper), assessed via polymer chain statistics of PE50 (CG) at 450 K. Results are reported with their standard deviations.

Model	κ_θ or $\kappa_{\theta,G96}$ [a]	$\langle R^2\rangle^{1/2}$ (nm)	$\langle R_g^2\rangle^{1/2}$ (nm)	$\langle(\theta-\theta_0)^2\rangle^{1/2}$ (rad)
this work	$\kappa_\theta = 8.5$	6.85 ± 0.17	2.71 ± 0.04	0.868 ± 0.005
conversion, Eq. (S5)	$\kappa_{\theta,G96} = 20.645$	6.81 ± 0.18	2.70 ± 0.04	0.865 ± 0.004
conversion, Eq. (S7)	$\kappa_{\theta,G96} = 83$	16.9 ± 2.14	5.31 ± 0.43	0.510 ± 0.018

[a] The angle force constant κ_θ is given in kJ mol^{-1} rad^{-2} and $\kappa_{\theta,G96}$ in kJ mol^{-1}. Conversion is performed from $\kappa_\theta = 8.5$ to $\kappa_{\theta,G96}$ using $\theta_0 = 180°$.

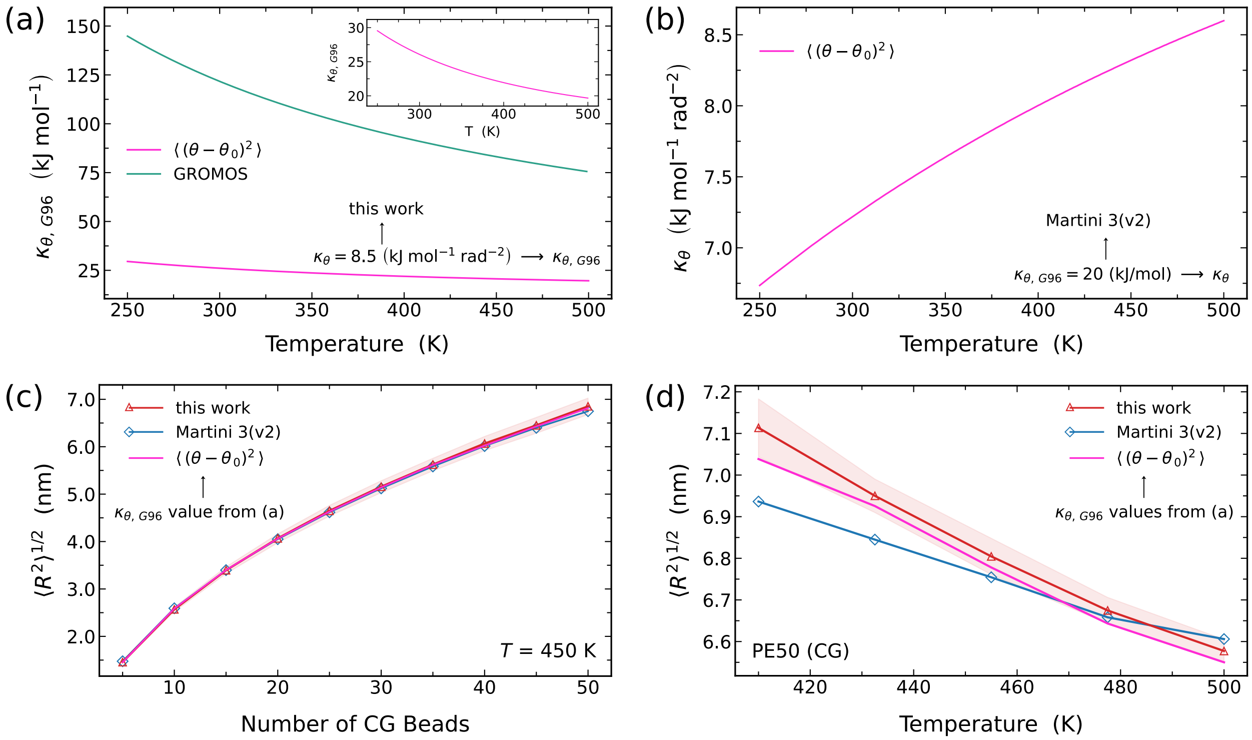

Figure S4: Conversion between angle force constants: (a) from κ_θ to $\kappa_{\theta,G96}$ and (b) from $\kappa_{\theta,G96}$ to κ_θ. Validation via the RMS end-to-end distance for the converted values in (a): (c) at 450 K across different degrees of polymerization and (d) for PE50 (CG) across different temperatures. Shaded region indicates standard deviation of our Martini-type PE model (this work).

IV Polymer Chain Statistics for PE25 (CG)

Figure S5 shows the temperature dependence of polymer chain statistics for PE50 (PE25 (CG)) in the mapped AA reference and the models presented in the main paper.

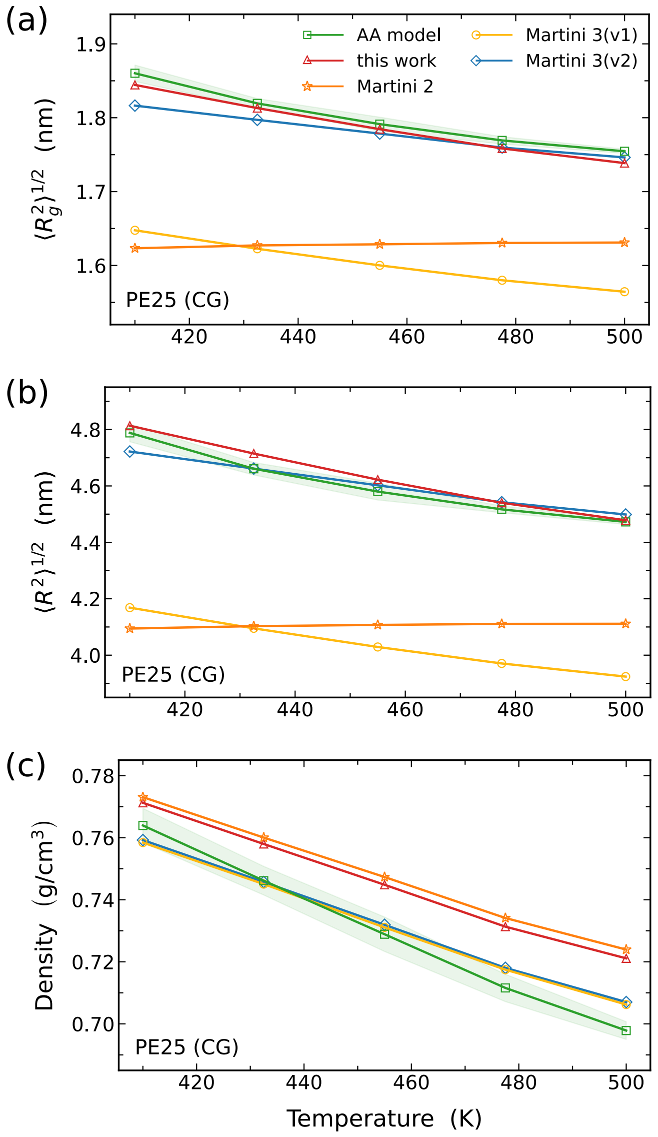

Figure S5: Polymer chain statistics for PE50 (PE25 (CG)) as a function of temperature for our Martini-type polyethylene (PE) model, existing Martini PE models, and the mapped AA reference. (a) RMS radius of gyration (Eq. (5) in the main paper), (b) RMS end-to-end distance (Eq. (4) in the main paper), and (c) density. Shaded region indicates standard deviation of the AA model; standard deviations of the coarse-grained PE models are similar and omitted for clarity.

V Interchain Radial Distribution Function of PE Melts

Figure S6 illustrates the interchain radial distribution function (RDF) for PE100 (PE50 (CG)) at 450 K for the mapped AA reference and the models presented in the main paper. The CG models preserve the characteristic packing length scale of the melt and the decay to bulk behavior, such that the positions of the RDF peaks remain close to those of the AA reference. In the AA model, local interchain packing arises from a distribution of conformations subject to steric constraints, whereas the CG representations smooth over these conformations, allowing more configurations at similar and slightly shorter separations. This results in higher short-range peaks and a slight inward shift of the RDF in the CG models. Overall, the CG models capture the melt structure of the AA reference.

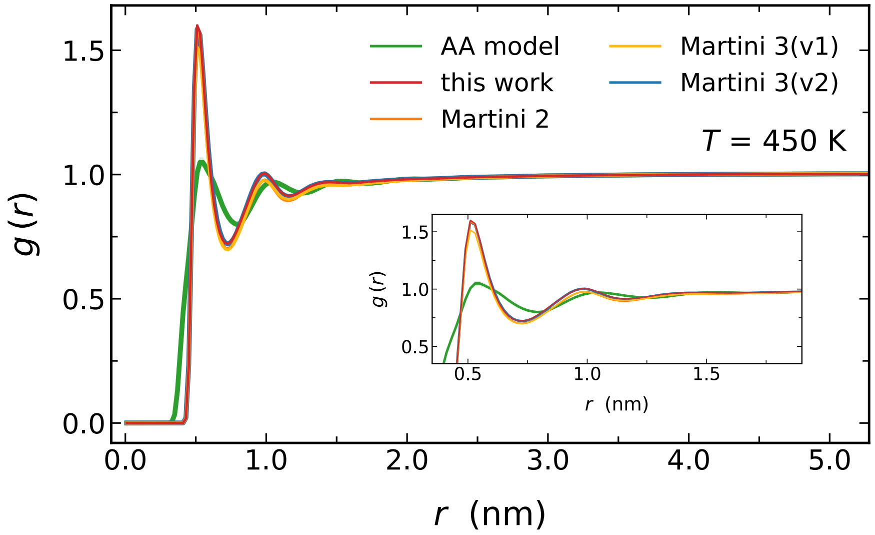

Figure S6: Interchain radial distribution function (RDF) for PE100 (PE50 (CG)) at 450 K (melt state). Shaded region indicates standard deviation of the AA model, which is roughly the same size as the line width; standard deviations of the coarse-grained PE models are similar and omitted for clarity.

VI Comparison Between Mapped and Unmapped AA Models

Figure S7 compares the mapped and unmapped AA references as functions of chain length (expressed as half the number of monomers for consistency with the CG mapping) and as functions of temperature for PE100. Deviations decrease with increasing chain length and remain nearly constant across temperatures, as the CG mapping is tied to chain length. The root-mean-square radius of gyration differs by less than 1% for PE chains with 20 monomers or more. The RMS end-to-end distance differs by about 2% for PE chains with 40 monomers and decreases to less than 1% for chains with 80 monomers or more.

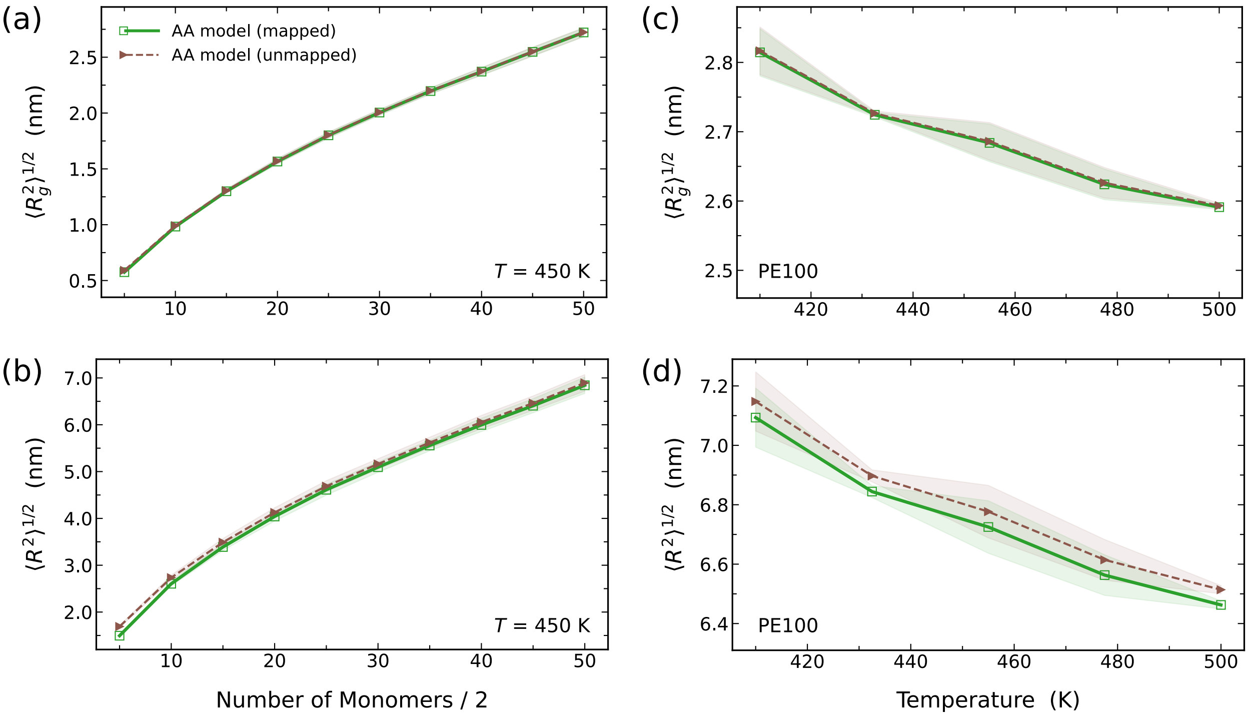

Figure S7: Polymer chain statistics for the mapped and unmapped AA references. Panels (a,b) as a function of half the number of monomers (corresponding to the CG mapping) at 450 K: (a) RMS radius of gyration (Eq. (5) in the main paper) and (b) RMS end-to-end distance (Eq. (4) in the main paper). Panels (c,d) as a function of temperature for PE100: (c) RMS radius of gyration and (d) RMS end-to-end distance. Shaded regions indicate standard deviations of both AA references.

VII Melting and Nucleation Behavior of the Martini-Type PE Model

We use the crystals from Sec. III.2 of the main paper to estimate the melting temperatures of PE50 (CG) and the corresponding AA model. Isothermal simulations are performed at 1 K intervals to identify the temperature at which the crystallinity χ decreases to zero. The crystallinity χ is defined in Sec. II.3.4 of the main text. The coarse-grained simulations are run for 3 μs, while the AA simulations are run for 300 ns. The hexagonal crystalline domains disappear at 381 ± 4 K for the coarse-grained model and at 433 ± 2 K for the AA model.

In molten-state simulations, we observe rapid homogeneous nucleation at 290 ± 5 K for the coarse-grained model and at 385 ± 5 K for the AA model.

VIII Nanoplastic Stability

As stated in Sec. III.3 of the main text, the nanoplastic-water system is first equilibrated at 280 K, heated to 310 K over 10 ns, and subsequently simulated for an additional 90 ns at 310 K. To assess the stability of the NPLs under these conditions, we report the time evolution of the radius of gyration R_g and equivalent spherical radius R over 1 μs in Fig. S8. These structural parameters are defined in Sec. III.3 of the main text. For both PE50 (CG) nanoplastics containing 25 and 50 chains, the structural parameters remain stable throughout the simulation, indicating that the NPLs reach stable configurations prior to membrane insertion.

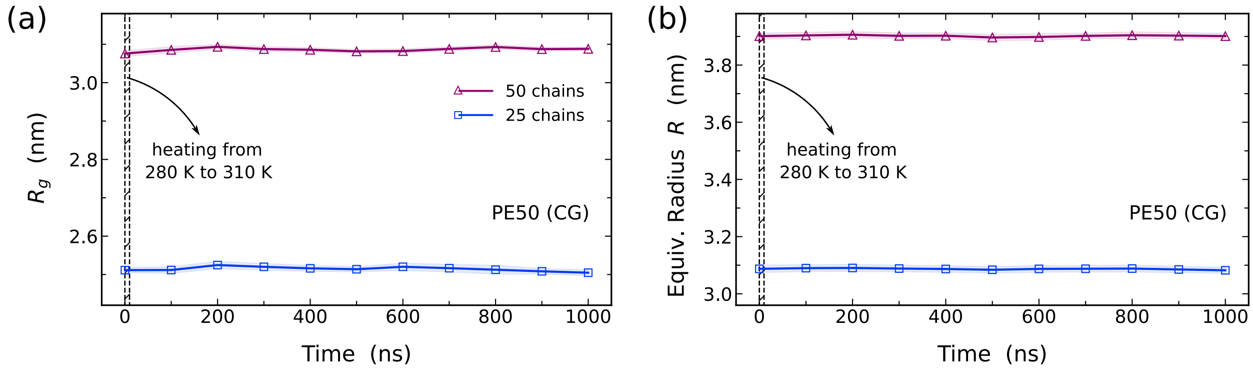

Figure S8: Nanoplastic structural parameters for PE50 (CG) with 25 and 50 chains over 1 μs prior to POPC membrane insertion. (a) Radius of gyration R_g and (b) equivalent spherical radius R (Sec. III.3 of the main text) as a function of time. Hatched regions indicate heating from 280 K to 310 K, while shaded regions represent the standard deviations of the curves.

References

(1) Duraes, A. D. S.; Zhang, W. Effects of Orientational and Conformational Ordering on Isotactic Polypropylene Crystallization. *Macromolecules* **2026**, *59*, 962–973, DOI: 10.1021/acs.macromol.5c03407.

(2) Strobl, G. Single Chain Conformations. In *The Physics of Polymers: Concepts for Understanding Their Structures and Behavior*; Springer Berlin Heidelberg: Berlin, Heidelberg, 2007; Chapter 2, pp 15–67, DOI: 10.1007/978-3-540-68411-4.

(3) Rubinstein, M.; Colby, R. H. *Polymer Physics*; Oxford University Press, 2003; Chapter 2, pp 49–96, DOI: 10.1093/oso/9780198520597.001.0001.

(4) Landau, L. D.; Lifshitz, E. M. *Statistical Physics*, 3rd ed.; Course of Theoretical Physics; Butterworth-Heinemann: Oxford, 1980; Vol. 5; Chapter 12, pp 333–400, DOI: 10.1016/C2009-0-24487-4, *translated from the Russian by* J. B. Sykes and M. J. Kearsley.

(5) Powell, M. J. D. An Efficient Method for Finding the Minimum of a Function of Several Variables without Calculating Derivatives. *Comput. J.* **1964**, *7*, 155–162, DOI: 10.1093/comjnl/7.2.155.

(6) Piessens, R.; de Doncker-Kapenga, E.; Überhuber, C. W.; Kahaner, D. K. *QUADPACK: A Subroutine Package for Automatic Integration*; Springer Series in Computational Mathematics; Springer-Verlag: Berlin, Heidelberg, 1983; DOI: 10.1007/978-3-642-61786-7.

(7) The GROMOS Software for (Bio)Molecular Simulation. *Volume 2: Algorithms and Formulae for Modelling of Molecular Systems*; BIOMOS b.v., Biomolecular Software: Zürich, Switzerland, 2023; Chapter 5 and 18, pp 25–29, 209–210, https://www.gromos.net/gromos11_pdf_manuals/vol2.pdf (accessed February 03, 2026).

(8) GROMACS development team. *GROMACS Documentation: Release 2023.3*; 2023;

Chapter 5, p 399, https://manual.gromacs.org/documentation/2023.3/manual-2023.3.pdf (accessed April 15, 2026).
